# Do 3^rd^ year medical students know how to use allergy and asthma devices?

**DOI:** 10.1186/1710-1492-8-S1-A27

**Published:** 2012-11-02

**Authors:** LE Meng, L Douglas, JA Seabrook, J Liem

**Affiliations:** 1 Schulich School of Medicine and Dentistry – Windsor, Western University, Canada; 2 Windsor Allergy Asthma Education Centre, Windsor, Ontario, Canada; 3 Department of Paediatrics, Western University, Canada; 4 Children’s Health Research Institute, Children’s Hospital, London Health Sciences Centre, Canada

## Introduction

It is expected that by the end of 4 years of medical training, medical students should be comfortable with the teaching and use of asthma and allergy devices. We sought to determine third-year medical students’ comfort levels in the use of these devices and evaluated the effectiveness of a medical device training seminar.

## Methods

65 third-year medical students participated in an asthma and allergy device training seminar provided by Windsor Allergy and Asthma Education Centre during their Pediatrics core rotation. The students’ comfort levels with the use of 7 devices were self-graded on a scale of 1-10 prior to training, and then again immediately after. Students’ interests in either medical or surgical specialties, presence of asthma and/or allergy conditions in the students, and stages of clinical training were collected. Mean comfort level scores before and after the training were compared using a paired t-test.

## Results

Prior to the seminar, mean comfort level scores ranged from 1.78 to 3.66 for each medical device. Scores were consistently low, regardless of the students’ interests in particular specialties. Mean scores ranged from 8.65 to 9.15 after the seminar, which represented a significant increase for every device (p<0.05).

## Conclusion

Third-year medical students were not comfortable with the use of asthma and allergy devices. A medical device training seminar increased the trainees' comfort level and should be considered as a regular part of the clinical training curriculum. Further study is warranted to determine whether the improved comfort level is retained at the end of 4-years of medical training.

